# A Review of the Advances in the Medical Management of Epilepsy Associated With Myoclonic Epilepsy With Ragged-Red Fibers (MERRF) Syndrome

**DOI:** 10.7759/cureus.82875

**Published:** 2025-04-23

**Authors:** Josef Finsterer

**Affiliations:** 1 Neurology Department, Neurology and Neurophysiology Center, Vienna, AUT

**Keywords:** antiseizure drugs, epilepsy, merrf, myoclonus, seizures

## Abstract

Myoclonic epilepsy with ragged-red fibers (MERRF) syndrome is a rare syndromic mitochondrial disorder in which epilepsy is one of the main phenotypic features. Although myoclonic seizures are most common in MERRF, several other seizure types (e.g., focal and generalized seizures with motor or nonmotor onset) have been reported. The literature search was conducted via PubMed and Google Scholar and covered the years 1966-2024. The author analyzes recent advances in treating epilepsy in MERRF with antiseizure medications (ASMs). Also discussed are the treatment of status epilepticus and stroke-like episodes (SLEs), and alternative methods of treating epilepsy. Treatment of epilepsy in MERRF depends on the phenotype (classic MERRF, MERRF-plus, overlaps, and SLEs), degree of progression, seizure types, type of epilepsy, seizure frequency, and the presence/absence of status epilepticus. ASMs and non-ASMs with a potentially mitochondrial toxic effect, as shown by clinical and experimental studies, should be administered with caution. MERRF patients should be closely monitored for epilepsy as the disease progresses, as new types of seizures or an increase in seizure frequency and intensity may occur. Recent advances suggest that myoclonic epilepsy responds most effectively to levetiracetam, benzodiazepines, and possibly zonisamide. If epilepsy is drug-resistant, alternative measures should be considered, as some of them may be very effective.

## Introduction and background

Primary mitochondrial disorders (MIDs) are genetic diseases caused by mutations in genes of the mitochondrial DNA (mtDNA) or nuclear DNA [1]. The phenotypic expression of these variants is highly heterogeneous and can range from asymptomatic cases to severe multisystem diseases with early death. MID phenotypes can be classified as syndromic or nonsyndromic depending on whether a constant and recurrent pattern of clinical manifestations is present. MIDs can occur at any age, but some of the MIDs preferentially begin in childhood, while others preferentially begin in adulthood. The most common syndromic MIDs include mitochondrial encephalopathy with lactic acidosis and stroke-like episodes (MELAS), Leigh syndrome, and maternally inherited diabetes and deafness syndrome. Rarer syndromic MIDs include Leber’s hereditary optic neuropathy (LHON), Kearns-Sayre syndrome, and myoclonic epilepsy with ragged-red fibers (MERRF) syndrome [1]. The most commonly affected organs are the brain, endocrine organs, and the heart. One of the most common clinical manifestations of cerebral involvement is epilepsy. Epilepsy in MIDs is explained by neuronal dysfunction caused by energy deficit in affected neurons and glial cells. The treatment of epilepsy in MIDs is based on classical and new antiseizure medications (ASMs), but due to frequent refractoriness to ASM therapy, alternative treatment options should also be considered.

MERRF is a rare syndromic, multisystem MID caused by variants in mtDNA genes and rarely nuclear genes [2]. These mutations manifest phenotypically as classic MERRF, MERRF plus, or MERRF overlap syndromes [3]. The classic MERRF phenotype is defined by the presence of the four canonical features: generalized epilepsy, myopathy with ragged red fibers, myoclonus, and ataxia. In addition to the canonical features, MERRF-plus patients can present with migraine, mental retardation, psychiatric disorders, dementia, stroke-like episodes (SLEs), optic atrophy, polyneuropathy, pigmentary retinopathy, hypoacusis, diabetes, hypothyroidism, short stature, cardiomyopathy, arrhythmias, vomiting, dysmotility, dysphagia, or lipomatosis. MERRF syndrome can also present as an overlap with other mitochondrial syndromes, such as MELAS syndrome, Leigh syndrome, LHON, or progressive external ophthalmoplegia [4]. The average age at onset is 45 years [2]. The prevalence of MERRF is unknown, but is estimated to be less than 1:100,000 [4]. MERRF is genetically heterogeneous, but it is established that the pathogenic variant m.8344A>G in MT-TK is responsible for approximately 80% of cases [5]. Other mutations manifesting as MERRF include the variants m.8356T>C, m.8363G>A, m.3243A>G, m.3255G>A, m.3291T>C, and variants in MTRNR1 and MTRNR2 [6]. As there is no specific or effective treatment for MERRF in general, only symptomatic measures can be taken.

One of the most common features of classic MERRF and MERRF plus is epilepsy. Epilepsy is one of the most common severe neurological disorders, affecting 51 million people worldwide in 2021 [7]. Epilepsy in MERRF most commonly manifests as myoclonic epilepsy. Myoclonic epilepsy in MERRF is classified as a subtype of progressive myoclonic epilepsy (PME) [8]. PMEs are characterized by a combination of myoclonic seizures and tonic-clonic seizures that are progressive (decline of motor skills, balance, and cognitive functions over time). PMEs include Lafora disease, Unverricht-Lundborg disease (Baltic myoclonus and epilepsy), neuronal ceroid lipofuscinosis (Batten disease), sialidosis, type-3 neuronopathic Gaucher disease, dentatorubral-pallidoluysian atrophy, myoclonus-renal failure syndrome, PME-ataxia syndrome, North Sea PME, myoclonus epilepsy and ataxia due to pathogenic variants in the potassium channel, and MERRF. MERRF differs from the other PMEs in its clinical presentation, including the involvement of multiple systems and SLEs in MERRF-overlap syndromes. The treatment of epilepsy in MERRF is challenging because the epilepsy is progressive and often only successful for a few months, and because some of the ASMs are potentially mitochondrion-toxic, which further worsens the course of epilepsy. This narrative review aims to provide an overview of the current state of progress in the pharmacological treatment of epilepsy in MERRF patients.

The data for this review were obtained by searching MEDLINE and Google Scholar for references to relevant articles. The following search terms were used to search the databases: "MERRF", "myoclonic epilepsy with ragged-red fibres", "juvenile myoclonic epilepsy", "MT-TK1", or "m.8344A>G" in combination with "epilepsy", "generalized epilepsy", "seizures", "antiepileptic drugs", "antiseizure drugs", "myoclonus", "convulsions", and "antiepileptic treatment". The results of the search were checked for potentially relevant studies using inclusion and exclusion criteria for the full texts of the relevant studies. In addition, the reference lists were searched for articles that were not found by the primary search. Randomized controlled trials, observational studies with controls, case series, case reports, and specific review articles were included. Editorials and letters to the editor were not included. Only articles reporting on specific ASM treatment, dosage, and duration of therapy for epilepsy in MERRF were included. Studies that only mentioned epilepsy and ASM in general terms were excluded. All articles that met these criteria and were published between 1966 and December 2024 were included. PubMed search for “MERRF” plus “epilepsy” yielded 606 hits, “MERRF” plus “seizures” 72 hits, “MERRF” plus “antiepileptic drugs” 17 hits, “MERRF” plus “antiseizure drugs” one hit, “MERRF” plus “myoclonus” 218 hits, “MERRF” and “convulsions” 69 hits, and “MERRF” plus “antiepileptic treatment” 18 hits. Of these, 39 articles were used for the review. The remaining works were found using other combinations of search terms.

## Review

Classification of epilepsy

The application of the International League Against Epilepsy (ILAE) framework for the classification of epilepsy begins with the determination of seizure type [9]. This determination is based on the assumption that clinicians have already made a definitive diagnosis of an epileptic seizure [9]. It is not intended as a diagnostic algorithm for distinguishing epileptic from nonepileptic events [9]. In some situations (e.g., in third world countries or rural areas), classification by seizure type may be the maximum possible level for the diagnosis of epilepsy, as there may be no access to electroencephalography (EEG), video-EEG, or cerebral imaging [9]. In other cases, there is simply too little information available to make a higher level diagnosis. This is the case, for example, when a patient has only had a single seizure [9].

Seizure types in MERRF

Various types of seizures have been described in MERRF patients. The most prevalent are myoclonic seizures. Myoclonic seizures are defined as myoclonus due to abnormal brain activity. Myoclonus is characterized by a rapid, alternating contraction and relaxation, twitching or jerking, of a muscle, usually lasting no longer than one to two seconds. It can be a single myoclonus, but it can also be several that occur within a short period of time. According to the 2017 ILAE classification of seizures, myoclonic seizures can have a motor or nonmotor onset (Table 1) [10]. In addition to generalized myoclonic seizures, focal myoclonic, focal atonic, focal sensory, generalized tonic-clonic, generalized atonic, generalized myoclonic-atonic, typical absences, or tonic-clonic seizures of unknown severity have also been reported in MERRF patients (Table 1) [5,11]. Status epilepticus has rarely been reported in MERRF, but may occur and should be considered, especially in patients with an inadequate response to ASM.

**Table 1:** Seizure types in MERRF according to the ILAE 2017 seizure classification MERRF: myoclonic epilepsy with ragged-red fibers; nr: not reported in MERRF; ILAE: International League Against Epilepsy

Seizure type	Reported in MERRF
Focal onset (preserved or impaired awareness)	
Motor onset	
Clonic	[12]
Tonic	nr
Myoclonic	[13]
Atonic	[13]
Hyperkinetic	nr
Spasms	nr
Automatisms	nr
Nonmotor onset	
Cognitive	nr
Emotional	nr
Sensory	nr
Behavioral	nr
Autonomic	nr
Generalized onset	
Motor onset	
Tonic-clonic	[14]
Tonic	nr
Clonic	nr
Myoclonic	[15]
Myoclonic-tonic-clonic	nr
Myoclonic-atonic	[13]
Atonic	[13]
Spasms	nr
Nonmotor (absences)	
Typical	[16]
Atypical	nr
Myoclonic	[16]
Eyelid clonus	nr
Unknown onset	[17]

Epilepsy types and epilepsy syndromes in MERRF

Epilepsy types and epilepsy syndromes described in MERRF follow the ILAE classification of epilepsies. According to the ILAE classification of epilepsies [9], four categories are distinguished. These include focal epilepsy, generalized epilepsy, combined generalized and focal epilepsy, and the unknown type of epilepsy [9]. MERRF patients predominantly have focal or generalized epilepsy or a combination of both. Focal and generalized seizures are not necessarily associated with a myoclonic component. At the syndromic level, epilepsy in MERRF is classified as PME with multisystem involvement.

Requirements for epilepsy treatment in MERRF

In order to optimally treat epilepsy in MERRF patients, several requirements should be met. First, it is important that the diagnosis has been genetically confirmed. Treating epilepsy based solely on the suspicion that a person has MERRF can be misleading in terms of general treatment, but particularly ASM treatment. Therefore, patients with a classic or MERRF-plus phenotype, as well as those with MERRF overlap, should undergo extensive genetic testing until a pathogenic variant associated with the phenotype is identified. Second, it is important that the phenotype is fully elucidated. It is crucial to know which organs or systems are affected, as the involvement of the liver or kidneys, for example, can be crucial for the choice of ASM. Therefore, patients with genetically confirmed MERRF require a thorough workup, not to miss subclinical or mildly manifesting organ disease. It is particularly important to clarify whether the phenotype includes SLE, as these are often associated with seizures, either as a trigger or as a complication. Although SLEs have been rarely reported in classic MERRF, they are common in MERRF/MELAS overlap syndromes. In MERRF patients with SLE, not only must seizures during SLE be treated but also the SLE itself by administering nitric oxide (NO) precursors such as L-arginine or L-citrulline. The third point is that for the optimal treatment of epilepsy in MERRF patients, it is crucial to accurately identify the types of seizures and epilepsy present in an affected individual. To achieve this goal, it may be helpful to examine these patients using video-EEG monitoring. The fourth point is that adherence to ASM treatment must be ensured either by the nursing staff, by the patient themselves, or by measuring ASM serum levels if available. Therefore, it must be clarified whether there is a mental disability that could potentially prevent the reliable use of the ASM.

Treatment of epilepsy in MERRF with ASM

There are no specific clinical drug trials for the treatment of epilepsy in MERRF patients. There are also no specific guidelines for the treatment of epilepsy in MERRF patients. However, general recommendations for the treatment of mitochondrial epilepsy have been published [18-22], which can be recommended as a guide for further action. In addition, expert opinions, case series, and case reports provide valuable guidance for orientation and the identification of the optimal individual treatment (Table 1). In addition to ASM treatment, most MERRF patients are treated empirically with “cocktails” of vitamins, antioxidants, and cofactors, including high-dose idebenone and L-carnitine [23,24].

The most commonly reported ASM for the treatment of epilepsy in MERRF is levetiracetam. In an uncontrolled study of 17 MERRF patients carrying the m.8344A>G variant, monotherapy with levetiracetam, clonazepam, valproic acid, or topiramate resulted in a stable disease or partial response in 16 patients after a follow-up period of one to four months [25]. A partial response was observed in four patients on monotherapy, with two in the levetiracetam group and two in the clonazepam group [25]. Ten patients remained stable on monotherapy, six in the clonazepam group, three in the levetiracetam group, and one in the topiramate group. Three patients had a progressive course of the disease. Twelve of the 17 patients were switched to dual therapy with levetiracetam plus clonazepam [25]. All patients who were switched to levetiracetam in combination with clonazepam showed a positive effect and good tolerability [25]. Eight of them also showed improved cognition on the mini-mental state examination and coordination on the Scale for the Assessment and Rating of Ataxia test [25]. The combination therapy was found to be significantly more effective for myoclonic seizures than the monotherapy [25]. However, levetiracetam or piracetam may also be ineffective in monotherapy in some cases. In a 71-year-old woman with MERRF due to the m.8344A>G mutation, myoclonic seizures responded only to piracetam [13]. Oxcarbazepine, levetiracetam, and lamotrigine were ineffective in this particular patient [13]. Valproic acid has only rarely been reported in MERRF patients or carriers of the m.8344A>G variant. In a 16-year-old boy with MERRF/Leigh overlap syndrome, generalized epilepsy began at the age of 14 and was successfully treated with valproic acid [26]. No severe side effects of treatment were reported in this particular patient [26]. Disadvantages of these case reports are that they may be subjective and biased, and lack rigor. Only a few studies reported a beneficial effect of clonazepam or zonisamide for myoclonic epilepsy in MERRF [23], but the positive effect of this treatment has not been well substantiated [23]. Perampanel or rufinamide could potentially be effective in the epilepsy of MERRF patients, as they have been used successfully in observational studies and post hoc analyses of patients with nonmitochondrial myoclonic epilepsy (Table 2) [27,28]. Other ASMs commonly used in patients with myoclonic epilepsy, but not specifically in MERRF, are valproate, phenobarbital, topiramate, or primidone, although phenobarbital should be given with caution [29]. Since most of the available ASMs have not been used in MERRF patients, there is a strong need to test ASM such as eslicarbazepine, phenobarbital, primidone, brivaracetam, seletracetam, padsevonil, rufinamide, perampanel, lacosamide, pregabalin, vigabatrin, tiagabine, felbamate, oxcarbazepine, ethosuximide, gabapentin, and K-bromide for myoclonic epilepsy (Table 2).

**Table 2:** Effect of ASM on epilepsy and myoclonus in MERRF syndrome AED: antiepileptic drugs; ASM: antiseizure medication; MERRF: myoclonic epilepsy with ragged-red fibers; nr: not reported; ↑↓: increase or reduction

AED	Acronym	Effect ↑↓ on seizure frequency or intensity	Effect on myoclonus	Reference
Valproic acid	VPA	Carnitine deficiency	↓	[15,30]
Carbamazepine	CBZ	nr	↑	[4,30]
Phenytoin	PHT	nr	↑	[4,30]
Phenobarbital	PB	nr	↓	[30]
Primidone	PRM	nr	↓	[29]
Oxcarbazepine	OXC	nr	↑	[30]
Ethosuximide	ESM	nr	↓	[31]
Benzodiazepines	NZP, CLZ	↓	↓	[15,23,30,32]
Levetiracetam	LEV	↓	↓	[30,33,34]
Lamotrigine	LTG	↓ or ↑	↓ or ↑	[30]
Gabapentin	GBP	nr	None or ↑	[30,35]
Topiramate	TPM	↓	↓ (as add-on)	[25,30]
Zonisamide	ZNS	↓	None	[15,23,30]
Tiagabine	TGB	nr	↑	[30,35]
Vigabatrin	VGB	nr	↑	[30,35]
Pregabalin	PGB	nr	↑	[30,35]
Lacosamide	LAC	nr	↓	[36]
Rufinamide	RFM	nr	↓	[37]
Perampanel	PER	nr	↓	[38]
Piracetam	PIR	↓	↓	[13,39]

Treatment of status epilepticus

Status epilepticus is a common feature of MERRF and should be treated according to guidelines with intravenous benzodiazepines such as lorazepam or midazolam together with levetiracetam and, if ineffective, phenytoin, fosphenytoin, or valproic acid [40]. Refractory status epilepticus should be treated with a continuous infusion of ASM such as midazolam, pentobarbital, thiopental, or propofol. Propofol should not be administered intravenously in children due to the risk of propofol infusion syndrome [40].

Treatment of SLEs

Although SLEs are more common in MELAS than in MERRF, they have occasionally been observed in MERRF patients. SLEs are often associated with seizures. There is currently no consensus or practical guidance on the most effective treatment for SLE. However, there is evidence that nitric oxide (NO) precursors, such as L-arginine or L-citrulline, may have beneficial effects, improving the symptoms of SLE and reducing its duration and severity. There are also reports that L-arginine prevents further SLEs [41]. An improvement in endothelial function could explain the effect of L-arginine. Authors who consider SLEs to be triggered solely by seizure activity recommend ASM for all SLE, regardless of whether paroxysmal activity is present on the EEG or whether the SLE is preceded or accompanied by a seizure.

Side effects and mitochondrial toxicity of ASMs

Some of the ASMs administered to MERRF patients may have specific side effects. In particular, several of them have been reported to exacerbate myoclonus. ASMs that may exacerbate myoclonus include phenytoin, carbamazepine, oxcarbazepine, phenytoin, vigabatrin, tiagabine, gabapentin, and pregabalin (Table 2) [4,29]. There is also a report showing that lamotrigine can exacerbate or even aggravate myoclonus [29]. There are also reports showing an increase in myoclonus with felbamate [42,43]. Some ASMs should be administered with caution in MERRF, as mitochondria are very sensitive to toxins and drugs that can impair various organelle functions [44]. Drugs that may be potentially mitochondrion-toxic include ASMs, which are nevertheless often recommended or used in the treatment of seizures in MERRF patients, as they have a strong seizure suppressive effect. Antiseizure drugs with an experimental (preclinical) mitochondrion-toxic effect include valproic acid, carbamazepine, phenytoin, and phenobarbital [5]. In animal models and cell cultures, valproic acid impairs mitochondrial function by reducing the activity of complex I and complex IV [45,46], and also affects various other mitochondrial functions [44]. Carbamazepine inhibits mitochondrial ATPase, thereby reducing mitochondrial respiration, energy output, and calcium handling [47]. Phenobarbital inhibits complex I activity and, thereby, energy production and other mitochondrial characteristics [44]. Phenytoin inhibits mitochondrial Na/K-ATPase and Mg-ATPase, which secondarily causes respiratory chain dysfunction and reduced energy production [48]. Non-ASMs that are known to be mitochondrion-toxic and may affect ASM treatment are antiretroviral antivirals (nucleoside reverse transcriptase inhibitors), cytostatics, and statins [49]. Other drugs that can be mitochondrion-toxic include anthracyclines, mitoxantrone, cyclophosphamide, cisplatin, fluorouracil, imatinib, bortezomib, trastuzumab, arsenic trioxide, cyclosporine-A, zidovudine, lamotrigine, glycosides, lidocaine, isoproterenol, nitroprusside, pivalic acid, cocaine, pesticides, cadmium, mycotoxins, cyanotoxins, and carbon monoxide [49]. Evidence of mitochondrial toxicity is derived from both experimental and clinical studies. For example, it is known that mitochondrial myopathy develops in association with statins in about 1% of cases. Carbamazepine, oxcarbazepine, and phenytoin have also been reported to exacerbate myoclonic episodes in MERRF patients [4]. Following these considerations, ASMs should be used with caution in MERRF patients, particularly if MERRF is caused by POLG1 variants [50].

Alternative antiepileptic treatments

In the event that epilepsy in MERRF becomes drug-resistant, alternative remedies should be considered. These include the Atkins diet, glucocorticoids, cannabidiol, N-acetyl cysteine, epilepsy surgery, vagus nerve stimulation, or deep brain stimulation [24]. Although most of these options have not yet been used in MERRF patients and are not evidence-based, clinicians and researchers should keep their applicability in mind as an off-label treatment. In a 16-year-old boy with MERRF who had weekly seizures and status epilepticus once a year, vagal nerve stimulation led to the disappearance of status epilepticus, and the frequency of seizures was significantly reduced [51].

Recommendations for epilepsy treatment in everyday clinical practice

In general, treatment of epilepsy in MERRF should be individualized because treatment depends on the phenotypic presentation and number and type of organs involved, as well as the degree of overlap with other mitochondrial syndromes that manifest with or without epilepsy. It is also important that MERRF patients be closely monitored for their epilepsy because the disease may progress and new phenotypic features or worsening of epilepsy may occur with increasing frequency or severity of seizures, new types of seizures may emerge, or SLEs may recur, and ASM can quickly become ineffective. MERRF patients should be monitored every three months by performing clinical status, blood count, renal function, liver function, and ASM levels, as well as an EEG. If seizures in MERRF patients do not respond to specific therapy, the ASM regimen should be modified and adjusted liberally, and alternative therapies such as cannabidiol, glucocorticoids, vagal stimulation, the Atkins diet, or invasive measures should be considered. Newer therapies for myoclonic epilepsy include deep brain stimulation and transcranial magnetic stimulation [52], which can lead to a 30%-40% reduction in seizure frequencies.

## Conclusions

The treatment of epilepsy in MERRF patients is challenging as it depends on numerous influencing factors, such as knowledge of the genetic background, phenotype (classic MERRF, MERRF plus, overlaps, MERRF spectrum disorders, and SLEs), degree of progression, type of seizures, types of epilepsy, seizure frequency, and the presence or absence of status epilepticus. The ASMs most commonly prescribed in patients with classic MERRF include levetiracetam and benzodiazepines (e.g., clonazepam). However, their dosage may vary depending on the individual needs of the patient and the treatment center. In addition, piracetam, benzodiazepines, zonisamide, topiramate, and possibly perampanel and rufinamide may be effective. ASMs with potentially mitochondrial toxic effects can also be administered, but with caution. It is recommended to monitor MERRF patients every three months using clinical examinations, blood tests, serum ASM levels, and EEG, as the disease may progress and new phenotypic features or worsening of epilepsy may occur with an increase in seizure frequency or severity or new seizure types. If epilepsy becomes drug-resistant in MERRF patients, alternative measures should be considered, as some of them can be very effective.
